# Stimulation of autophagy promotes functional recovery in diabetic rats with spinal cord injury

**DOI:** 10.1038/srep17130

**Published:** 2015-11-24

**Authors:** Kai-liang Zhou, Yi-fei Zhou, Kai Wu, Nai-feng Tian, Yao-sen Wu, Yong-li Wang, De-heng Chen, Bin Zhou, Xiang-yang Wang, Hua-zi Xu, Xiao-lei Zhang

**Affiliations:** 1 Department of Orthopaedics, The Second Affiliated Hospital of Wenzhou Medical University, Wenzhou, Zhejiang Province, China; 2 Zhejiang Provincial Key Laboratory of Orthopaedics, Wenzhou, Zhejiang Province, China

## Abstract

In this study we examined the relationship between autophagy and apoptosis in diabetic rats after spinal cord injury (SCI), also we determined the role of autophagy in diabetes-aggravated neurological injury *in vivo* and *in vitro*. Our results showed that diabetes decreased the survival of neurons, promoted astrocytes proliferation, increased inflammatory cells infiltration and inhibited functional recovery after SCI. Diabetes was shown to confer increased activation of apoptotic pathways, along with an increase in autophagy; similar effects were also observed *in vitro* in neuronal PC12 cells. Treatment with rapamycin, an autophagy activator, partially abolished the adverse effect of diabetes, suggesting that diabetes may enhance neurological damage and suppress locomotor recovery after SCI, in addition to its effects on apoptosis and autophagy. In contrast, further stimulation of autophagy improved neurological function via inhibition of apoptosis. These results explained how diabetes exacerbates SCI in cellular level and suggested autophagy stimulation to be a new therapeutic strategy for diabetic SCI.

Spinal cord injury (SCI) remains a serious health problem worldwide, affecting ~273,000 people in the United States alone, with almost 12,000 new cases occurring every year^1^. SCIs can result in significant loss of motor and sensory functions, leading to a wide range of disabilities. These effects can often be exacerbated by other co-morbidities, such as the metabolic disorder diabetes mellitus, which has been associated with structural and functional changes in both the peripheral and central nervous system^2^. Previous studies have shown that diabetes impairs functional improvement after spinal cord injury in animals and humans^3^,^4^; however, few studies have examined the mechanism by which spinal cord injury is aggravated in diabetic patients.

The pathology of SCI is divided into two stages: the primary injury and the secondary injury. The primary injury is the mechanical impact afflicted directly on the spine, whereas, the secondary injury is a complex cascade of molecular events including disturbances in ionic homeostasis, local oedema, ischemia, focal haemorrhage, oxidative stress and inflammation^5^, with apoptosis playing a vital role in the progressive degeneration of the injured spinal cord^6^. As apoptosis has been shown to be accelerated in the injured spinal cord of diabetic rats, these effects may contribute to the delayed recovery from SCI often seen in diabetic patients^7^.

Autophagy is the process by which cells degrade cytosolic macromolecules and organelles in lysosomes, and is generally considered to be a survival tactic used to protect against metabolic stress^8^. As autophagy acts as a specific type of programmed cell death, it shares many of the pathways and regulatory mechanisms of apoptosis^9^. Autophagy is known to play an important role in a variety of degenerative conditions, including Alzheimer’s disease, diabetes, and ageing. A recent study using a rat model of diabetic intervertebral disc degeneration demonstrated strong induction of both autophagy and apoptosis^10^, suggesting a mechanistic link between autophagy and diabetes. Besides, autophagy has been shown to be activated in animal model of traumatic spinal cord injury^11^. We therefore hypothesised that the activation of autophagy plays an important role in cases of diabetic SCI.

Numerous studies suggested a strong link between autophagy and neurotrauma. It has been demonstrated that autophagy is activated in many neurotrauma models, including cerebral ischemia, traumatic head injury, and spinal cord injury^12^. Besides, the role of autophagy in neurotrauma is likely to depend on conditions characteristic of a given disease. On the one hand, the promotion of autophagy was considered to provide a neuroprotective function in models of neonatal hypoxia-ischemia-induced brain injury, closed head injury and spinal cord injury^13^,^14^. On the other hand, several other studies reported that autophagy also causes cell death (autophagic cell death) in focal cerebral ischemia^15^and neonatal cerebral ischemia^16^. However whether the function of autophagy is protective or detrimental for neural tissue in diabetes mellitus after SCI remains to be elucidated.

Here, we examined the effects of diabetes mellitus on autophagy, apoptosis, and neurological damage (increased inflammation, astrocytes proliferation and neuronal loss) in the context of a spinal cord injury. Rats were examined with and without rapamycin, a classical autophagy agonist widely applied *in vivo* and *in vitro*^17^,^18^, to better define the role of autophagy in acute SCI in diabetic rats, and to identify the internal mechanism involved in apoptosis. Finally, the mechanism of action underlying pathology in diabetic SCI rats is further verified via *in vitro* analysis in PC12 cells.

## Results

### Successful induction of SCI in a murine model of Type 1 diabetes

**Figure 1:**
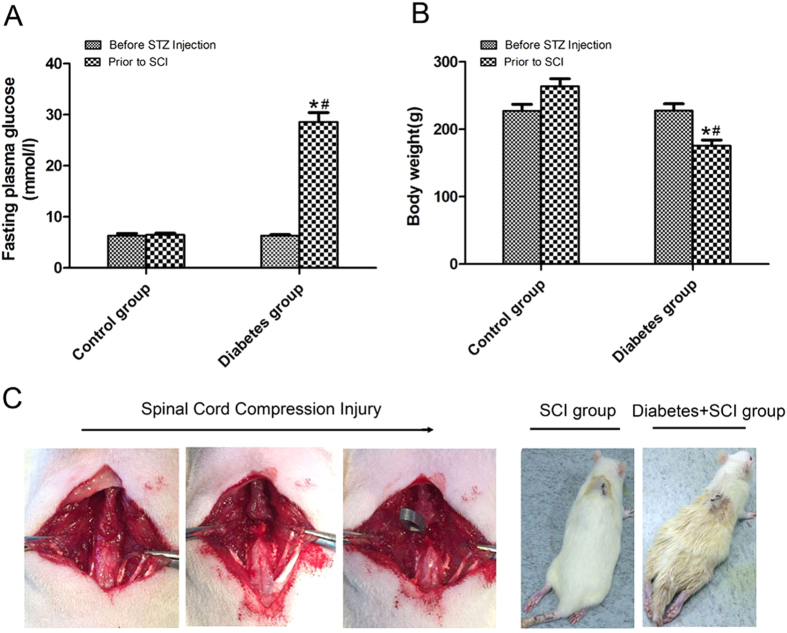
Induction of SCI in a murine model of Type 1 diabetes (**A**) The fasting plasma glucose of rats in control group and diabetes group before STZ injection and prior to SCI. (**B**) The body weight of rats in the two groups before STZ injection and prior to SCI. (**C**) The procedure of the SCI model from left to right, as shown by black arrowhead. Values are expressed as the mean ± SEM. n = 32 per group. *P < 0.05 vs control group prior to SCI, ^#^*P* < 0.05 vs diabetes group before STZ injection.

No differences in fasting plasma glucose (FPG) levels or mean body weight of rats were detected between diabetes and control groups before streptozotocin (STZ) injection. Four weeks after the induction of diabetes (prior to SCI), the mean weight of the diabetic animals was 175.67 ± 8.47 g and FPG levels were 28.57 ± 1.82 mmol/L, compared with a mean weight of 263.83 ± 11.41 g, and FPG levels of 6.47 ± 0.36 mmol/L and 5.42 ± 0.23 mmol/L, respectively for controls (*P* < 0.05 for both; Fig. 1A,B). Following induction of SCI, rats in both the SCI and diabetes + SCI groups showed dramatic and bilateral hind limb paralysis with no movement at all or only slight movements of a joint in the first few hours post-injury (Fig. 1C). Twelve diabetic rats died within the first week after SCI, for a mortality rate of 18.75%; two deaths were recorded in the control group.

### Diabetes decreases neuronal survival, promotes astrocytes proliferation, increases inflammation and inhibits functional recovery after SCI

**Figure 2:**
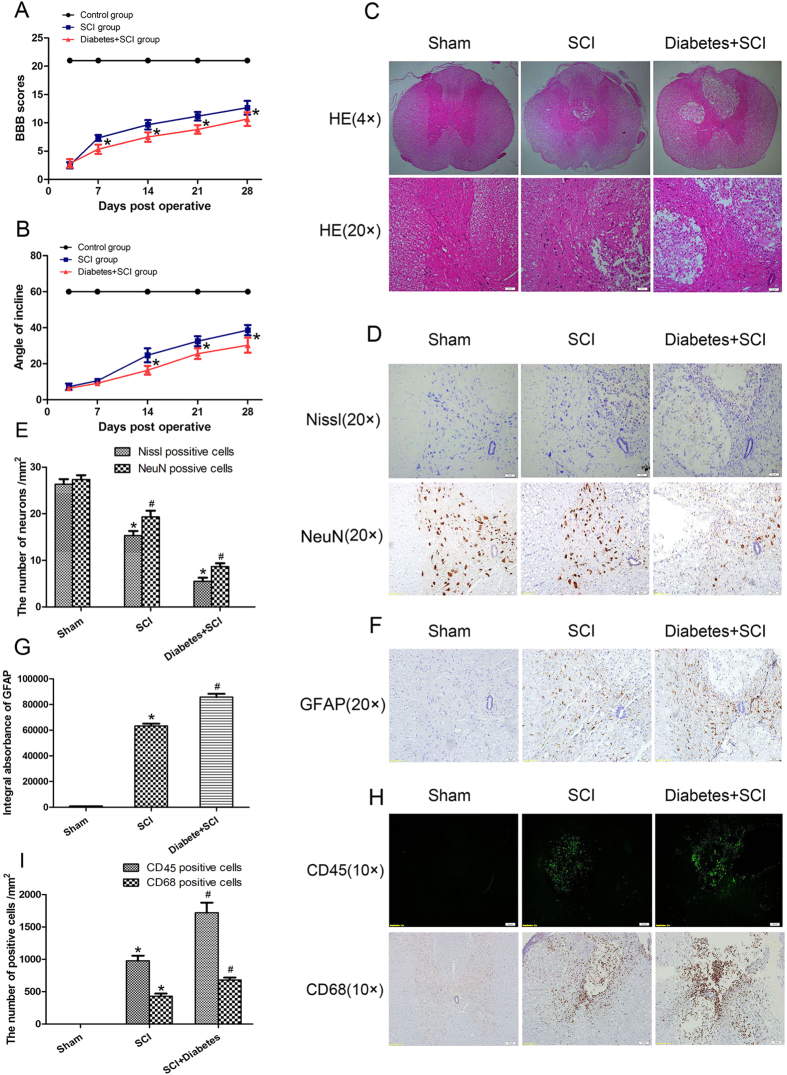
Diabetes decreases neuronal survival, promotes astrocytes proliferation, increases inflammation and inhibits functional recovery after SCI. (**A**) The Basso, Beattie and Bresnahan (BBB) scores of rats in Sham group, SCI group and Diabetes + SCI group at 3, 7, 14, 21 and 28 days after contusion. (**B**) The inclined plane test scores of three groups at 3, 7, 14, 21 and 28 days after contusion. (**C**) HE staining of the injured spinal cords in three groups to evaluate the histological change. (**D**) Nissl staining and Immunohistochemistry (for NeuN) to assess the loss of neurons in Sham, SCI and Diabetes + SCI groups, respectively. (**E**) The bar graph shows the quantitative Nissl/NeuN positive cells in spinal cord lesions. (**F**) Immunohistochemistry (for GFAP) to assess the the proliferation of astrocytes in Sham, SCI and Diabetes + SCI groups, respectively. (**F**) The bar graph shows the quantitative IA value of GFAP in three groups. (**G**) Immunofluorescence for CD45 and Immunohistochemistry for CD68 to determine the inflammation in spinal cord lesions in Sham, SCI and Diabetes + SCI groups, respectively. (**I**) The bar graph shows the quantitative CD45/CD68 positive cells in three group. Values are expressed as the mean ± SEM, n = 5 per group. *P < 0.05 versus the Sham group, ^#^*P* < 0.05 versus the SCI group.

To evaluate the effects of diabetes on locomotor recovery after SCI, Basso, Beattie, and Bresnahan (BBB) scores and inclined plane test results were measured for 4 weeks. No significant differences in BBB scores were detected between the SCI and diabetes + SCI groups 3 days after SCI (*P *> 0.05; Fig. 2A); however, diabetes + SCI rats BBB scores were significantly lower than that of SCI controls at 7, 14, 21, and 28 d after contusion (*P* < 0.05). Similarly, the inclined plane test scores were consistently lower in diabetes + SCI rats at 14, 21, and 28 d after injury (*P* < 0.05; Fig. 2B). These data suggest that diabetes may suppress functional improvement of locomotor activity after SCI. Histomorphology differences between the sham control, SCI, and diabetes + SCI groups were analysed by H&E staining 7 days after contusion (Fig. 2C). Progressive destruction of the dorsal white matter and central grey matter was evident in SCI rats relative to the sham control group, indicative of spinal cord injury. Comparisons between the SCI and diabetes + SCI groups revealed significant adverse effects, including increased infiltration of inflammatory cells and a larger cavity involving the lateral funiculi, dorsal and central gray matter in the diabetes + SCI group. Direct analysis of neuronal survival in the spinal cord was investigated using Nissl staining and immunohistochemistry [for NeuN, a common neuronal marker] 7 days after injury (Fig. 2D). The positive cells of Nissl and NeuN significantly decreased in the spinal cord lesions in SCI rats, with further reductions seen in the diabetes + SCI group (*P* < 0.05; Fig. 2E). To assess the proliferation of astrocytes in the injured spinal cord, Immunohistochemistry staining for glial fibroblast acid protein (GFAP) was performed in each group (Fig. 2F). As is shown in Fig. 2G, diabetes mellitus significantly increased the IA value of GFAP expression in spinal cord after SCI (*P* < 0.05). To determine the inflammation in spinal cord lesions, Immunofluorescence staining for CD45 (a common leukocyte marker) and Immunohistochemistry staining for CD68 (a macrophages marker) was performed (Fig. 2H). In the image of Fig. 2I, we found that diabetes significantly increased the number of CD45/CD68 positive cells in spinal cord lesions (*P* < 0.05). These results show that diabetes decreased neuronal survival, promoted astrocytes proliferation, increased inflammation and inhibited functional recovery after SCI.

### The adverse effect of diabetes is related to apoptosis in SCI rats

**Figure 3:**
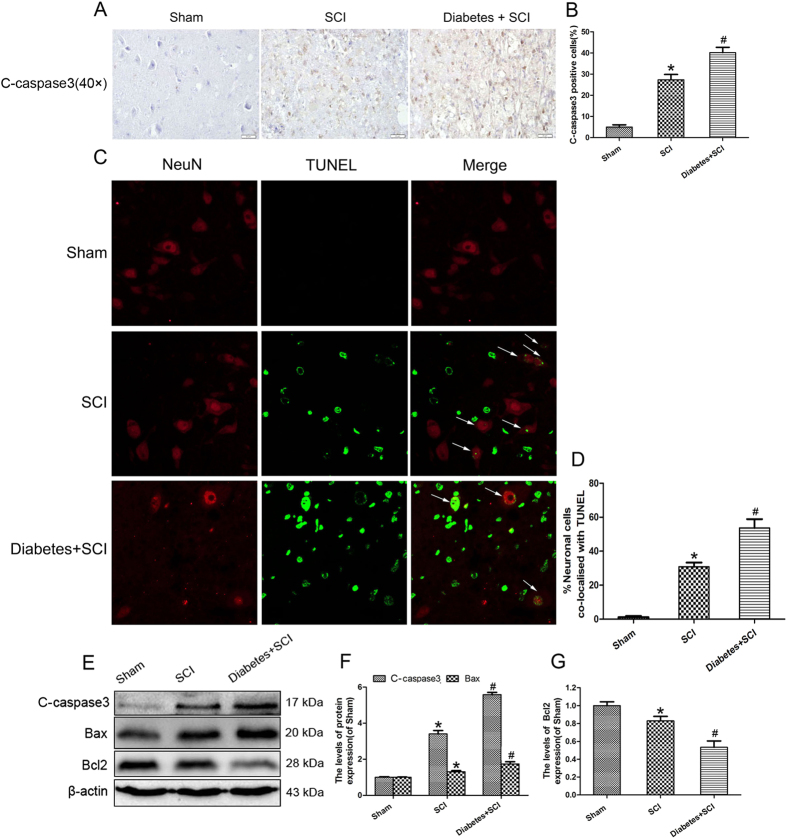
Increased level of apoptosis in spinal cord lesions in diabetic rats. (**A**) Immunohistochemistry for C-caspase3 in the three groups on the 7^th^ day after SCI(original magnification×400). (**B**) The bar graph shows the quantitative C-caspase3 positive cells in spinal cord lesions.(**C**) Double staining for NeuN(red)/TUNEL(green) of sections from the injured spinal cord in Sham group, SCI group and Diabetes + SCI group. (**D**) The bar graph shows the quantitative percentage of the apoptotic neurons in spinal cord lesions. (**E**) Protein expressions of C-caspase3, Bcl2 and Bax in the spinal cord segment at the contusion epicenter. β-actin was used as the loading control and for band density normalization. (**F,G**) The optical density analysis of C-caspase3, Bcl2 and Bax proteins. Values are expressed as the mean ± SEM, n = 5 per group. *P < 0.05 versus the Sham group, ^#^*P* < 0.05 versus the SCI group.

To assess the role of apoptosis in the injured spinal cord, Immunohistochemistry staining for C-caspase3 and double staining for NeuN/TUNEL were performed in each group. Immunohistochemical analysis of diabetes-induced apoptosis in SCI revealed significant increases in the number of C-caspase 3 positive cells in diabetes + SCI rats compared with that of SCI rats (*P* < 0.05; Fig. 3A,B). Double staining for NeuN (red)/TUNEL (green) was performed to observe the apoptosis in neurons of injured spinal cord (Fig. 3C). As is shown in Fig. 3D, the percentage of the apoptotic neurons significantly increased in the spinal cord lesions in SCI rats, with further increments seen in the diabetes + SCI group (*P* < 0.05). Increased expression of C-caspase 3, Bax, and Bcl2 were also detected by Western blotting, revealing strong induction of both C-caspase-3 and Bax after SCI contusion (Fig. 3E). Further increases in both C-caspase 3 and Bax were detected in the diabetes + SCI groups relative to the SCI group 7 days after contusion (*P* < 0.05; Fig. 3F); the reverse was seen for Bcl2, with progressively lower levels seen in the SCI and diabetes + SCI groups relative to controls (*P* < 0.05; Fig. 3G). Taken together, these data suggest a direct link between the adverse effects of diabetes and apoptosis in SCI rats.

### Activation of autophagy in diabetic rats after SCI

**Figure 4:**
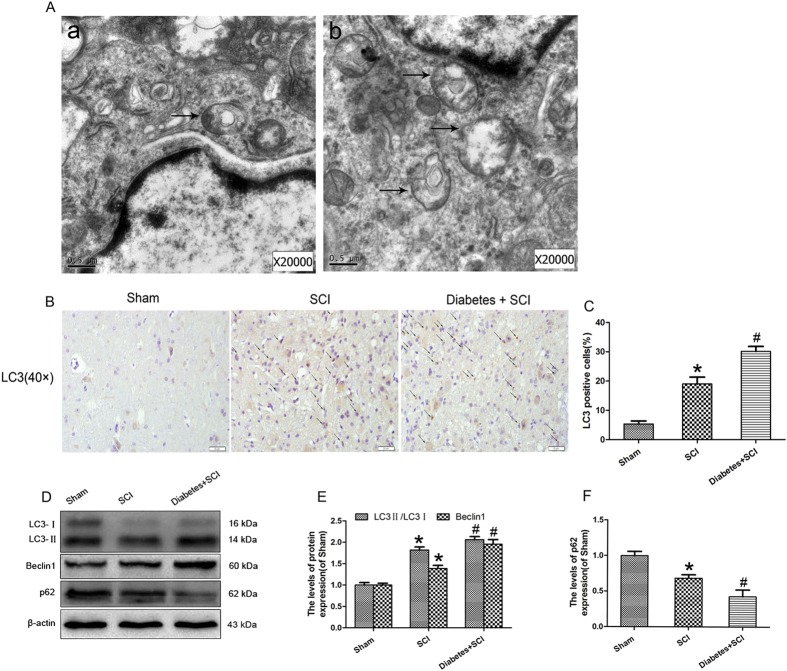
Increased level of autophagy in spinal cord lesions diabetic rats. (**A**)Transmission electron microscopy shows the autophagosomes (black arrowheads), and the scale bars indicate 0.5 μm. a: SCI group b: Diabetes + SCI group. (**B**) Immunohistochemistry for LC3 punctuated dots (black arrowheads) in Sham, SCI and Diabetes + SCI groups at 7 days after spinal cord injury(original magnification ×400). (**C**) The bar graph shows the quantitative LC3 punctuated dots positive cells in spinal cord lesions. (**D**) Protein expressions of LC3II/I, Beclin1 and p62 in the spinal cord segment at the contusion epicenter. β-actin was used as the loading control and for band density normalization. (**E,F**) The optical density analysis of LC3II/I, Beclin1 and p62 proteins. Values are expressed as the mean ± SEM, n = 5 per group. *P < 0.05 versus the Sham group, ^#^*P* < 0.05 versus the SCI group.

We used transmission electron microscopy (TEM) to observed autophagosomes, double membrane structures containing parts of cytoplasmic organelles, in the spinal cords of SCI and diabetes + SCI rats (Fig. 4A). To molecularly confirm the induction of autophagy, we used Immunohistochemistry to detect the autophagosomal marker LC3II in the cytoplasm of affected cells. LC3II-positive cells were shown to be increased in spinal cord lesions, relative to controls, with further induction seen in the injured spinal cords of diabetic rats (*P* < 0.05; Fig. 4B,C). Protein expression was confirmed by Western blot analysis using antibodies targeting LC3II/LC3I and Beclin1, with results broadly consistent with that of Immunohistochemistry (*P* < 0.05; Fig. 4D,E). Despite clear evidence of autophagosomes, detection of autophagosomes alone does not indicate activation of the autophagic process. Autophagy is a dynamic mechanism of degradation of damaged cellular organelles and long-lived proteins. The protein p62 is a substrate of the autophagic process, and its expression is considered a marker of autophagic flux^19^. Expression of p62 in diabetic rats after SCI was detected by Western blot analysis, revealing levels much lower than that of SCI rats and controls (*P* < 0.05; Fig. 4D,F). These results indicated that autophagy activation plays a role in the exacerbation of disease in diabetic rats after spinal cord injury.

### The adverse effects of diabetes in SCI are inhibited by autophagy agonist rapamycin

**Figure 5:**
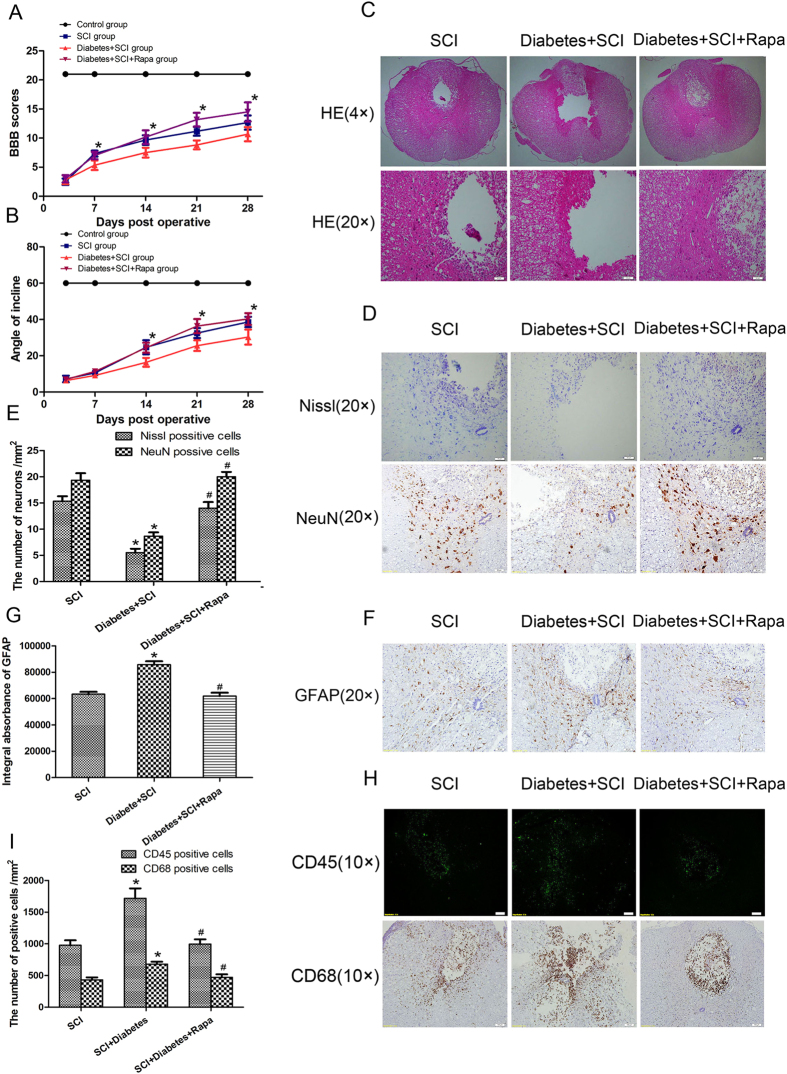
Stimulating autophagy by rapamycin promotes SCI functional recovery in diabetic rats. (**A**) The Basso, Beattie and Bresnahan (BBB) scores of rats in SCI group, Diabetes + SCI group and Diabetes + SCI +Rapa group at 3, 7, 14, 21 and 28 days after contusion. (**B**) The inclined plane test scores of these three groups at 3, 7, 14, 21 and 28 days after contusion. (**C**) HE staining of the injured spinal cords in three groups to evaluate the histological change. (**D**) Nissl staining and Immunohistochemistry (for NeuN) to assess the loss of neurons in SCI, Diabetes + SCI, Diabetes + SCI + Rapa groups, respectively. (**E**) The bar graph shows the quantitative Nissl/NeuN positive cells in spinal cord lesions of three groups. (**F**) Immunohistochemistry (for GFAP) to assess the the proliferation of astrocytes in SCI, Diabetes + SCI, Diabetes + SCI + Rapa groups, respectively. (F) The bar graph shows the quantitative IA value of GFAP in three groups. (**G**) Immunofluorescence for CD45 and Immunohistochemistry for CD68 to determine the inflammation in spinal cord lesions in SCI, Diabetes + SCI, Diabetes + SCI + Rapa groups, respectively.(**I**) The bar graph shows the quantitative CD45/CD68 positive cells in three group. Values are expressed as the mean±SEM, n = 5 per group. *P < 0.05 versus the SCI group, ^#^*P* < 0.05 versus the Diabetes + SCI group.

To determine the effects of autophagy activation during SCI recovery in diabetic rats, rapamycin, a classical autophagy agonist, was used to treat diabetic rats after SCI. BBB scores and angle of incline test results revealed significant improvements in movement functional recovery in diabetic SCI rats following rapamycin treatment relative to diabetes + SCI alone (Fig. 5A,B). HE staining revealed significant reductions in the damage to the dorsal white matter and central grey matter following rapamycin treatment in the diabetes + SCI group, with concomitant decreases in infiltration of inflammatory cells and a smaller cavity in lesion site (Fig. 5C). The effect of rapamycin on the survival of neurons in diabetic spinal cord was investigated using Nissl staining and immunohistochemistry (for NueN) 7 days after injury (Fig. 5D). Staining revealed lower loss of positively stained neurons in the lesions of rapamycin-treated diabetic rats, compared to the diabetes + SCI group (*P* < 0.05; Fig. 5E). Immunohistochemistry (for GFAP) results revealed significantly decreased the IA value of GFAP expression in diabetic SCI rats following rapamycin treatment relative to diabetes + SCI alone ((*P* < 0.05; Fig. 5F,G). The effect of rapamycin on inflammation in diabetic spinal cord was investigated using Immunofluorescence (for CD45) and Immunohistochemistry (for CD68) 7 days after injury (Fig. 5H). As is shown in Fig.5I, rapamycin significantly reduced the number of CD45/CD68 positive cells in diabetic SCI (*P* < 0.05). Taken together, these results provide that stimulation of autophagy increases neuronal survival, suppresses astrocytes proliferation, inhibits inflammation and promotes functional recovery of diabetes after SCI.

### Further increased autophagy by rapamycin reduces the level of apoptosis in diabetic rats after SCI

**Figure 6:**
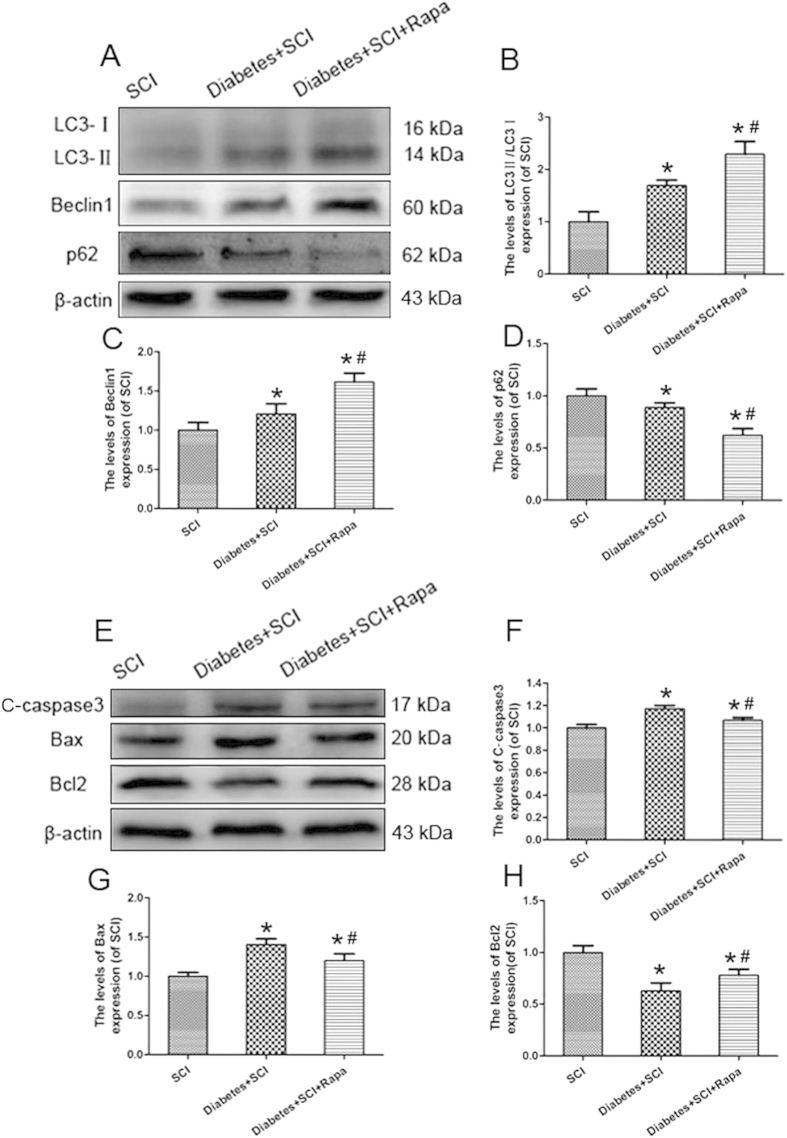
Rapamycin inhibits apoptosis and reduces neuron death in diabetic rats after SCI. (**A**) Protein expression of LC3II/I, Beclin1 and p62 of rats in SCI, Diabetes + SCI and Diabetes + SCI + Rapa groups, respectively. β-actin was used as the loading control and for band density normalization. (**B–D**) The optical density analysis of LC3II/I, Beclin1 and p62. (**E**) Protein expressions of cleaved caspase3, Bcl2 and Bax in the spinal cord segment at the contusion epicenter. β-actin was used as the loading control and for band density normalization. (**F–H**) The optical density analysis of C-caspase3, Bax and Bcl2. Values are expressed as the mean ± SEM, n = 5 per group. *P < 0.05 versus the SCI group, ^#^*P* < 0.05 versus the Diabetes + SCI group.

The expression levels of apoptosis-related proteins (C-caspase 3, Bax, and Bcl2) and autophagy-associated proteins (LC3-II/LC3-I, Beclin1, and p62) were evaluated to address the mechanism of autophagy induction in diabetic SCI rats. Rapamycin treatment was shown to significantly increase the LC3-II/LC3-I ratio and Beclin1 expression in the diabetes + SCI group (*P* < 0.05; Fig. 6A – C); significant decreases were seen for p62 (Fig. 6A,D). In contrast, both C-caspase 3 and Bax levels were decreased in diabetic SCI rats following rapamycin treatment; Bcl2 expression was also reduced in the rapamycin-treated group (*P* < 0.05; Fig. 6E,F,H). These results suggest that rapamycin-induced autophagy inhibits apoptosis in diabetic SCI rats, indicating a potential neuroprotective role for autophagy in cases of SCI.

### Up-regulation of autophagy suppresses high-glucose-aggravated apoptosis in an *in vitro* oxidative damage model

**Figure 7:**
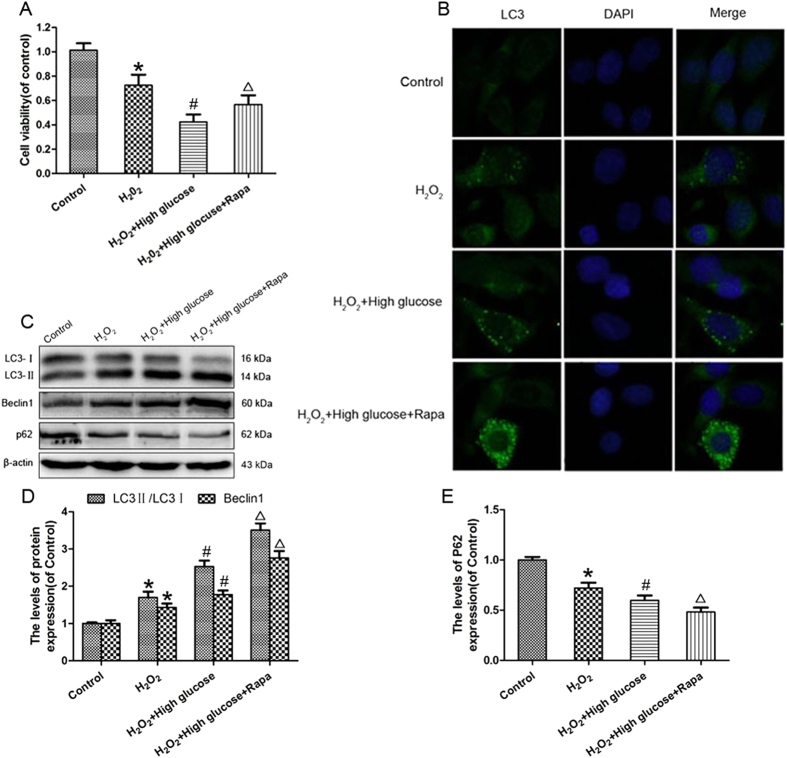
Rapamycin inhibits high glucose induced cell death by autophgay stimulation *in vitro*. (**A**) The CCK-8 results of PC12 cells in the Control, H_2_O_2_ model, and H_2_O_2_ model cells treated with high glucose and H_2_O_2_ model cells treated with high glucose compound with rapamycin. (**B**) Immunofluorescence staining results of LC3(green blot),where the nuclei are labeled with DAPI(blue). (**C**) The protein expressions of LC3II/LC3I, Beclin1 and p62 in PC12 cells of these four groups. β-actin was used as the loading control and for band density normalization (**D,E**) The optical density analysis of LC3II/LC3I, Beclin1 and p62 proteins in four groups. *P < 0.05 versus Control group, ^#^*P* < 0.05 versus H_2_O_2_ group, ^△^*P* < 0.05 versus H_2_O_2_ + High glucose. Rapa, rapamycin ; H_2_O_2_, hydrogen peroxide.

Hyperglycaemia plays a major role in the development of diabetes-associated diseases. Thus, high glucose was used to simulate the hyperglycaemic condition of diabetes *in vitro*. To further test our hypothesis that hyperglycaemia-induced autophagy contributes to neuroprotection *in vitro*, PC12 cells were subjected to oxidative damage via treatment with H_2_O_2_, and then incubated in the presence of high glucose concentrations with or without rapamycin. In CCK-8 assays, cell viability was dramatically inhibited following H_2_O_2_ treatment in the presence of high glucose concentrations, which was significantly attenuated in the presence of rapamycin (*P* < 0.05; Fig. 7A). Next, the effect of high glucose concentrations on autophagy was evaluated using immunofluorescence. Increased levels of LC3-II puncta in the cytoplasm of H_2_O_2_-treated PC12 cells were seen in the presence of high glucose concentrations (Fig. 7B); the number of LC3-II puncta in the high glucose + H_2_O_2_ group was further increased following the addition of rapamycin. High glucose concentrations significantly increased the LC3-II/LC3-I ratio and Beclin1 expression in H_2_O_2_-treated PC12 cells, which was further enhanced by the addition of rapamycin (*P* < 0.05; Fig. 7C,D); the opposite effect was seen for p62 (Fig. 6C,E). These findings suggest that activation of autophagy may play a role in neuroprotection in the presence of high glucose concentrations.

**Figure 8:**
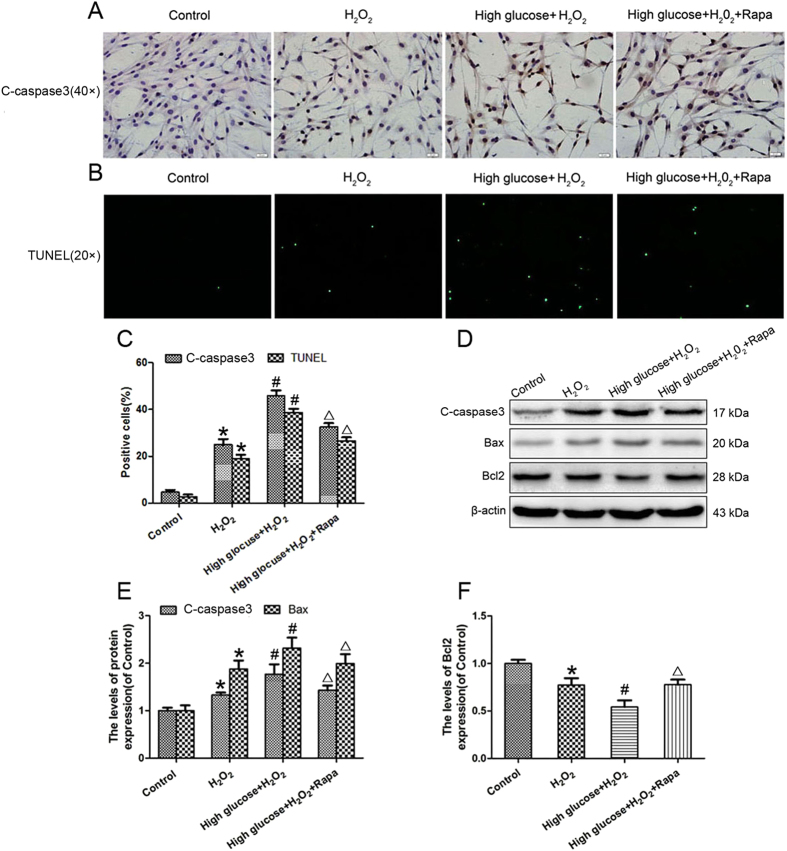
Rapamycin inhibits high glucose induced apoptosis *in vitro*. (**A**) Immunocytochemistry for C-caspase3 in PC12 cells of Control, H_2_O_2_, H_2_O_2_ + high glucose and H_2_O_2_ + high glucose + rapamycin groups, respectively (original magnification ×400). (**B**) The PC12 cells were collected and stained with TUNEL(original magnification ×200). (**C**) The bar graph shows the quantitative C-caspase3 and TUENL positive cells in four groups. (**D**) Protein expressions of C-caspase3, Bcl2 and Bax in the PC12 cells. β-actin was used as the loading control and for band density normalization.(**E,F**) The optical density analysis of C-caspase3, Bcl2 and Bax proteins in the four groups. *P < 0.05 versus Control group, ^#^*P* < 0.05 versus H_2_O_2_ group, ^Δ^*P* < 0.05 versus H_2_O_2_ + High glucose.

The molecular mechanism underlying the protective effects of autophagy *in vitro* was also connected with the inhibition of apoptosis. The number of TUNEL-positive cells was lower in high glucose + H_2_O_2_ cells treated with rapamycin relative to controls (*P* < 0.05; Fig. 8B,C). Similar levels of C-caspase 3 positive cells were detected by Immunohistochemistry, consistent with the TUNEL staining results (Fig. 8A,C). Increases in the expression of both C-caspase 3 and Bax in response to high glucose were attenuated following addition of rapamycin (*P* < 0.05; Fig. 8D,E); down-regulation of Bcl2 was also alleviated by rapamycin application (*P* < 0.05; Fig. 8D,F). Taken together, these data further confirm the neuroprotective role of autophagy activation in combination with inhibition of apoptosis in PC12 cells.

## Discussion

Serious spinal cord injuries often result in lifelong disability and enormous economic costs. Vertebral impact injuries, such as those caused by traffic accidents, sports injuries, and other physical traumas, are not uncommon. In cases of substantial spinal cord injury, the initial trauma is followed by a long period of secondary damages, characterised by persistent oxidative stress, inflammation, necrosis and apoptosis^20^,^21^. Similar molecular events are thought to play a key role in the pathophysiological degeneration of the injured spinal cord in diabetic rats, with diabetes-associated increases in apoptosis in the spinal cord significantly inhibiting recovery from spinal cord injury^4^,^7^. The data presented here strongly support these observations, with upregulation of apoptosis seen in diabetic rats following spinal cord injury. Previous studies have shown that Bcl2 exerts an anti-apoptotic effect^22^, while Bax participates in the induction of apoptosis^23^; activation of C-caspase 3 is seen as a hallmark of apoptotic cell death, acting as the final executor of apoptosis^24^. Western blot analyses revealed higher levels of both C-caspase 3 and Bax in the diabetes + SCI group relative to the SCI group, whereas expression of Bcl2 declined. Furthermore, Immunohistochemistry (for C-caspase 3) showed significant increases in the number of apoptosis-positive cells in the spinal cord lesions of diabetic rats. Double staining (for NeuN/TUNEL) also revealed that diabetes up-regulated the level of apoptosis in neurons from spinal cord lesions. Previous studies found that the inflammatory response^25^, astrocytic gliosis^26^ and neurons loss^20^ are three common indicators to reflect neurological damage of spinal cord injury. In our study, diabetic SCI revealed worse neurological damage, including increased inflammation, proliferation of astrocytes and neurons loss in lesion site. Based upon these data we conclude that STZ-induced diabetes significantly increased neurological damage and suppressed locomotor recovery after SCI in rats, due to increases in apoptosis.

Autophagy is a process of self-digestion whereby the cell degrades long-lived proteins and organelles as a means of generating the building blocks necessary to sustain cellular function^27^. While often considered solely as a response to starvation, autophagy has been shown to protect against pathologies, including neurodegenerative disorders^28^, infections^29^, inflammatory diseases^30^, and cancer^31^. Here, we show strong activation of both autophagy and apoptosis in the spinal cord lesions of diabetic rats, compared with non-diabetic controls. While the exact relationship between autophagy and apoptosis is not fully understood, previous studies have shown a direct relationship between autophagy activation and inhibition of apoptosis^13^,^32^. The reverse scenario is also true, with the inhibition of autophagy associated with increases in apoptosis, which may contribute to neuronal damage in neurodegenerative disease^33^. However, this inverse regulation is not uniform across all cell types, with autophagy shown to play an important role in apoptosis in bystander CD4 T lymphocytes^34^. Similarly, Grishchuk *et al.*^35^ found that Beclin 1-independent autophagy is an important contributor to both caspase-dependent and -independent components of neuronal apoptosis. In our model of diabetic SCI, we treated diabetic SCI rats with the autophagic agonist rapamycin to further determinate the relationship between autophagy and apoptosis. In the rapamycin treatment group, Bcl2 expression was increased in relation to the diabetes + SCI group, whereas the expressions of both C-caspase 3 and Bax declined, indicating that over-activation of autophagy inhibited the rate of apoptosis in diabetic SCI. In addition, rapamycin also reduced the degree of neuronal loss, and promoted functional recovery. Taken together, these results suggest that diabetes upregulated autophagy, increased apoptosis, and delayed locomotor recovery in spinal cord injury. Furthermore, stimulation of autophagy reduced neurological damage and promoted locomotor recovery via inhibition of apoptosis in diabetic SCI rats.

In the model described here, diabetes was induced via injection of a single large dose of STZ, a compound which specifically kills pancreatic β cells, mimicking the pathology of Type 1 diabetes^36^,^37^. These STZ-treated rats exhibited significant hyperglycaemia, in addition to other important co-morbidities, including hyperphagia, polydipsia, and weight loss. The resulting diabetic rats exhibit worse functional recoveries than that of their healthy counterparts when subjected to the same level of SCI. Hyperglycaemia is a major contributing factor to the development of diabetes-related diseases. Several biochemical events have been associated with hyperglycaemia, including glucose-mediated increases in reactive oxygen species, as well as advanced glycation of important functional and structural proteins^38^, suggesting that hyperglycaemia is a key factor in the pathophysiological changes and lag in function recovery in our diabetic SCI model. To further examine hyperglycaemic-mediated autophagy and apoptosis *in vitro*, PC12 cells were treated with H_2_O_2_ to cause oxidative damage, and then treated with high levels of glucose with or without rapamycin. The result showed that high glucose concentrations activated both apoptosis and autophagy in damaged PC12 cells, with glucose-induced apoptosis inhibited by the upregulation of autophagy.

As with all research, this work was not without limitations. One of the most significant limitations of this research is that the STZ-induced model of diabetes is reflective primarily of Type 1 diabetes, and therefore does not accurately recreate all aspects of the more common Type 2 diabetes. Second, since diabetic SCI rats have a higher mortality rate, the time course over which pathologies arise in the diabetic SCI model has not been established.

In conclusion, STZ-induced diabetes significantly exacerbated neurological damage and suppressed locomotor recovery after spinal cord injury in rats, in part through the enhanced activation of apoptosis. While increases in both autophagy and apoptosis were found to inhibit recovery from spinal cord injury in diabetic rats, further stimulation of autophagy improved the neurological function via inhibition of apoptosis. These data suggest a potential therapeutic strategy by which stimulation of autophagy may be beneficial for improving recovery from spinal cord injury in diabetic patients.

## Materials and Methods

### Animals

Adult female Sprague-Dawley rats (220–250 g) were purchased from Wenzhou Medical University (SCXK [Zhe] 2005–0019). Care and use of all animals conformed to the Guidelines set forth by the Chinese National Institutes of Health, with relevant study protocols approved by the Animal Care and Use Committee of Wenzhou Medical University (wydw2012–0079). Efforts were made to reduce the number of animals used in this study, and to minimise their suffering.

### Cell culture and treatments

PC12 cells were purchased from the Cell Storage Centre of Wuhan University (Wuhan, China), and maintained at 37 °C in a humidified atmosphere containing 5% (v/v) CO_2_. Cells were cultured in Dulbecco’s modified Eagle’s medium (DMEM; Gibco, Invitrogen, Grand Island, NY) supplemented with heat-inactivated 10% (v/v) foetal bovine serum (Gibco, Invitrogen, Grand Island, NY), and antibiotics (100 units/mL penicillin, 100 μg/mL streptomycin). PC12 cells were maintained in DMEM supplemented with 4.5 mg/mL glucose, according to the manufacturer’s instructions. High-glucose medium was defined as DMEM containing a threefold increased concentration of supplemental glucose (13.5 mg/mL). Cells were culture in DMEM for 24 h, transferred to fresh DMEM or high-glucose DMEM for 12 h, and then treated with H_2_O_2_ (Sigma; 100 μmol/L) for 2 h to induce oxidative injury. To further assess the effects of autophagy activation on high-glucose-induced injury, cells were treated with 100 nmol/L rapamycin (Sigma-Aldrich, St. Louis, MO, USA) for 2 h. Afterwards, cells were divided into four groups: controls, H_2_O_2_, H_2_O_2_ + high glucose, and H_2_O_2_ + high glucose + rapamycin. Cells were then analysed using a cell counting kit-8 (CCK-8; Dojindo Co, Kumamoto, Japan), immunofluorescence, immunocytochemistry, and Western blot. All experiments were performed in triplicate.

### Cell viability assay

Cell viability was assayed using the CCK-8 kit according to the manufacturer’s instructions. In brief, PC12 cells were plated on 96-well plates at a density of 10^5^ /mL, and incubated in DMEM at 37 °C for 24 h. Cells were then treated with H_2_O_2_, H_2_O_2_ + high glucose, or H_2_O_2_ + high glucose + rapamycin. Following incubation, cells were washed with PBS, 100 mL DMEM +10 μL CCK-8 solution were added to each well, followed by incubation for a further 2 h. The absorbance of the wells at 450 nm was then measured using a microplate reader.

### Immunocytochemistry

PC12 cells were plated on slides in a six-well plate and treated with H_2_O_2_, H_2_O_2_ + high glucose, or H_2_O_2_ + high glucose + rapamycin, as described above. For immunocytochemistry staining, samples were fixed in 4% (w/v) paraformaldehyde for 15 min and permeabilised with 0.1% (v/v) PBS-Triton X-100 for 30 min. To block the activity of endogenous peroxidase, the slides were incubated in 3% H_2_O_2_ for 15 min at room temperature. Cells were then blocked in 10% (v/v) bovine serum albumin in PBS for 1 h, and incubated with a primary antibody against cleaved caspase 3 (C-caspase 3; 1:400; Cell Signaling Technology, Danvers, MA) overnight at 4 °C. The next day, slides were washed and incubated with an horseradish peroxidase-conjugated secondary antibodies in PBS for 1 h, developed with DAB, and counterstained with haematoxylin. Finally, images were captured at ×400 magnification under a light microscope (Olympus, Tokyo, Japan). The C-caspase3 positive cells were automatically counted at eight randomly selected fields per sample by using the Image-Pro Plus (IPP) software, version 6.0 (Media Cybernetics, Rockville, MD).

### Immunofluorescence

To determine the LC3 activity and distinguish the CD45 positive cells, sections were permeabilised with 0.1% (v/v) PBS-Triton X-100 for 30 min. After blocking in 10% (v/v) bovine serum albumin in PBS for 1 h, sections were incubated at 4 °C overnight with a primary antibody against LC3 (1:200; Cell Signal Technology, Danvers, MA) and CD45(1:200, Abcam, Cambridge, MA, USA). After that, slides were washed 3 × 10 min at room temperature, and incubated with fluoresceinisothiocyanate (FITC)-conjugated goat anti-rabbit IgG (1:200) antibody for 1 h at room temperature. Images were captured under a fluorescence microscope (Olympus, Tokyo, Japan). Finally, the CD45 positive cells were automatically counted at eight randomly selected fields from lesion site per sample per sample by using the IPP software.

### Animal model of Type 1 diabetes

Sprague-Dawley rats were housed in a specific pathogen-free (SPF) room with a 12 h light/dark cycle and provided regular food and water for 1 week prior to any experimental procedure. One hundred and fifty rats were randomly divided into two groups: (1) control group, and (2) diabetes group. After fasting for 12 h, 70 rats in the diabetes group were intraperitoneally (i.p.) injected with 1% (w/v) streptozotocin (STZ) 60 mg/kg (Sigma-Aldrich, St. Louis, MO, USA) dissolved in citrate buffer (pH 4.0-4.4); the remaining 70 rats were fasted for 12 h and treated with a vehicle citrate buffer (1 mL/kg) as a control. All rats were given *ad libitum* access to the normal diet and tap water after injection. The fasting plasma glucose (FPG) of rats was measured in the tail blood with an autoanalyser (Roche, Mannheim, Germany). One week after STZ injection, FPG levels were tested, with levels >16.7 mmol/L considered indicative of successful Type 1 diabetes induction. FPG levels and body weights were measured for all animals both before injection, as well as 7, 14, 21, and 28 days after injection. Six rats in the diabetes group died within 28 days after injection.

### Animal model of SCI and drug administration

Within the control group, 70 rats were randomly divided into either the sham control or SCI group. Meanwhile, 64 diabetic rats were randomly divided into the diabetes + SCI or diabetes + SCI + rapamycin group. Rats in the SCI (n = 35), diabetes + SCI group (n = 32), and diabetes + SCI + rapamycin (n = 32) groups were subjected to induced SCI 4 weeks after STZ injection. Rats were anaesthetised by administration of 2% (w/v) pentobarbital sodium (40 mg/kg, Solarbio Science & Technology, Beijing, China) via intraperitoneal injection. There are 13 pairs of ribs in SD rats. As is well known, the T13 vertebrae is connected with the 13th ribs. And the 13th ribs and T13 vertebrae were determined in X-ray images (Fig. S1A), which were taken by a animals digital X-ray machine (Kubtec Model XPERT.8; KUB Technologies Inc.). After that, the T9 vertebrae was located via the locating pin before the procedure of SCI (Fig. S1B). Afterwards, the vertebral column was then exposed, and a laminectomy carried out carefully at the T9 vertebrae. The vertebrae were then subjected to a crushing injury via compression with a vascular clip (15 g force; Oscar, China) for 1 min to expose the spinal cord (Fig. 1C). Rats in the sham control group (n = 35) were subjected to the same surgical procedure with the spinal cord was exposed for 1 min, but did not undergo the compression injury. After SCI, rats underwent manual urinary bladder emptying five times daily until bladder function returned. Cefazolin sodium (50 mg/kg/day, i.p.) was administrated to prevent bacterial infection. Rapamycin (Sigma-Aldrich, St. Louis, MO, USA) was dissolved in DMSO (25 mg/mL) and further diluted in saline for intraperitoneal injection. Following spinal cord compression injury, rats in the diabetes + SCI + rapamycin group were treated with 1 mg/kg/day, while rats in the remaining groups were injected with the equivalent volume of vehicle for 7 days. All rats received daily rehabilitation, including passive mobilisation of the paralysed legs five times daily. Following completion of the trial, rats were euthanised using an overdose of pentobarbital sodium on day 7, with the exception of 20 rats, which were used to assess locomotion recovery.

### Locomotion recovery assessment

The Basso, Beattie, and Bresnahan (BBB) locomotion rating scale^39^ was used to assess locomotion recovery. BBB tests were conducted 3, 7, 14, 21 and 28 d after SCI; rats were laid on the floor and crawling ability was observed for 5 min. Outcome measures were obtained in a blinded fashion by independent examiners and averaged. In brief, BBB scores range from 0 points, which is indicative of complete paralysis, to 21 points which indicates normal locomotion. The inclined plane test^40^ was also performed to assess locomotion recovery of all rats in each time point. In brief, all rats were tested in two positions (right side or left side up) on a testing apparatus (i.e., a board covered with a rubber mat containing horizontal ridges spaced 3 mm apart). For each position, the maximum angle at which a rat could maintain its position for 5 s without falling was recorded and averaged to obtain a single score for each rat.

### Hematoxylin and Eosin (H&E) staining and Nissl staining

Rats (n = 5 per group) were re-anaesthetised with 2% (w/v) pentobarbital sodium (40 mg/kg, i.p.) and perfused with normal saline, followed by addition of 4% (w/v) paraformaldehyde in phosphate-buffered saline 7 days after surgery. Spinal cord segments (1 mm in length), that spanned a 3 mm length in the spinal cord centred at the epicentre were collected and fixed in 4% (w/v) paraformaldehyde for 24 h. Next, samples in each group were embedded in paraffin for transverse paraffin sections. The paraffin sections (4 μm thick) mounted on poly-L-lysine-coated slides for histopathological examination by H&E staining. Meanwhile other sections were incubated in 1% (w/v) Cresyl violet for Nissl staining and observed under a light microscope (Olympus, Tokyo, Japan). The Nissl positive cells were automatically counted at eight randomly selected fields from grey matter area per sample by using the IPP software.

### Immunohistochemistry

Rat spinal cord tissues (1 mm segments; n = 10 per group) were excised and fixed, as before. All samples were embedded in paraffin for either coronal or transverse paraffin sections, respectively. Paraffin sections (4 μm thick) were mounted on poly-L-lysine-coated slides for immunohistochemistry; transverse paraffin sections (for NeuN, GFAP and CD68) and coronal paraffin sections (for C-caspase3 and LC3)were deparaffinised with xylene and rehydrated through graded ethanol. After washing, the sections were blocked with 3% (v/v) H_2_O_2_ and treated with 10.2 mmol/L sodium citrate buffer for antigen retrieval for 20 min at 95 °C. After blocking in 10% (v/v) bovine serum albumin dissolved in PBS for 30 min, sections were incubated with antibodies against NeuN (1:200, Abcam, Cambridge, MA, USA), GFAP(1:500; 1:200, Abcam, Cambridge, MA, USA), C-caspase 3 (1:500; Cell Signal Technology, Danvers, MA), CD68 (1:200; Bio-Rad Laboratories, Inc., Hercules, CA))or LC3 (1:500; Cell Signal Technology, Danvers, MA) overnight at 4 °C. Next, sections were incubated with horseradish peroxidase-conjugated secondary antibodies for 2 h at 37 °C, developed with DAB, and counterstained with haematoxylin. Images were captured under a light microscope (Olympus, Tokyo, Japan), The integral absorbance (IA) values of GFAP was calculated and the positive cells of NeuN, C-caspase3, CD68 and LC3 were counted automatically at eight randomly selected fields from lesion site per sample by using the IPP software.

### TdT-mediated dUTP biotin nick-end labelling (TUNEL)

TUNEL was used to detect DNA fragmentation due to apoptotic signalling cascades *in vivo* and *in vitro*. Slides of PC12 cells were washed with distilled water, incubated with Proteinase K for 20 min at 37 °C, and analysed using the *In Situ* Cell Death Detection Kit (Roche Molecular Biochemicals) according to the manufacturer’s instructions. Images were visualised using a fluorescence microscope (Olympus, Tokyo, Japan) at 200× magnification, and the TUNEL positive cells were automatically counted at eight randomly selected fields from slide per sample by using the IPP software.

### Double staining of NeuN and TUNEL

To identify DNA fragmentation in neurons, the transverse sections at 7d after SCI were double-stained for TUNEL and NeuN (a neurons marker). Sections were permeabilised with 0.1% (v/v) PBS-Triton X-100 for 30 min. After blocking in 10% (v/v) bovine serum albumin in PBS for 1 h, sections were incubated at 4 °C overnight with a primary antibody against NeuN (1:100, Abcam, Cambridge, MA, USA). After that, slides were washed 3 × 10 min at room temperature, and incubated with FITC-conjugated goat anti-rabbit IgG (1:200) antibody for 1 h at room temperature. Then, Sections were washed 3 × 10 min at room temperature again and analysed using the *In Situ* Cell Death Detection Kit (Roche Molecular Biochemicals) according to the manufacturer’s instructions. Images were visualised using a fluorescence microscope (Olympus, Tokyo, Japan). The percentage of TUNEL positive neurons was automatically calculated at eight randomly selected fields from lesion site per sample by using the IPP software.

### Transmission electron microscopy (TEM)

After fixation in 2.5% (w/v) glutaraldehyde overnight, spinal cord tissues were post-fixed in 2% (v/v) osmium tetroxide and blocked with 2% (v/v) uranyl acetate. Following dehydration in a series of acetone washes, tissues were embedded in Araldite for coronal sections. Semi-thin section and toluidine blue staining were performed for observation of location. Finally, ultra-thin sections of at least three blocks per sample were cut and observed using a Hitachi TEM.

### Western blot analysis

Total proteins from spinal cord tissues and PC12 cells were purified using protein extraction reagents. Rat spinal cord segments (8 mm; containing the injury epicentre) (n = 5 per group) were dissected on day 7 following SCI. The equivalent of 60 μg protein were separated on a 12% (w/v) gel and transferred onto a PVDF membrane (Bio-Rad Laboratories, Hercules, CA, USA). After blocking with 5% (w/v) non-fat milk for 2 h, the membranes were incubated with the following antibodies: Bax, C-caspase 3, Bcl2, Beclin1, p62 (1:1000; Cell Signaling Technology; Danvers, MA, USA), LC3 (1:500; Cell Signaling Technology; Danvers, MA, USA), and β-actin (1:200; Santa Cruz Biotechnology). Next, the membranes were incubated with a goat-anti-rabbit secondary antibody for 2 h at room temperature, and bands detected using the enhanced chemiluminescence (ECL) kit (PerkinElmer, Waltham, MA, USA). Band intensity was quantified using the Image Lab 3.0 software (Bio-Rad).

### Statistical analysis

Statistical analyses were carried out using the SPSS.20 statistical software. All values are presented as the means ± standard error of the mean (SEM). Statistical evaluation of the data was performed by one-way ANOVA followed by a post hoc comparison test using the LSD (equal variances assumed) or Dunnett’s T3 (equal variances not assumed) method. P values < 0.05 were considered to indicate statistical significance.

## Additional Information

**How to cite this article**: Zhou, K.-l. *et al.* Stimulation of autophagy promotes functional recovery in diabetic rats with spinal cord injury. *Sci. Rep.* **5**, 17130; doi: 10.1038/srep17130 (2015).

## Supplementary Material

Supplementary Figure S1
