# Supplementary material for: Stimulation of autophagy promotes functional recovery in diabetic rats with spinal cord injury
Source: Sci Rep. 2015 Nov 24;5:17130. doi: 10.1038/srep17130 (PMC4657088; doi:10.1038/srep17130)
Supplement: Supplementary Figure S1 [file srep17130-s1.doc]

**Supporting information**

# Stimulation of autophagy promotes functional recovery in diabetic rats with spinal cord injury

Kai-liang Zhou, Yi-fei Zhou, Kai Wu, Nai-feng Tian, Yao-sen Wu, Yong-li Wang, De-heng Chen, Bin Zhou, Xiang-yang Wang, Hua-zi Xu*&Xiao-lei Zhang*


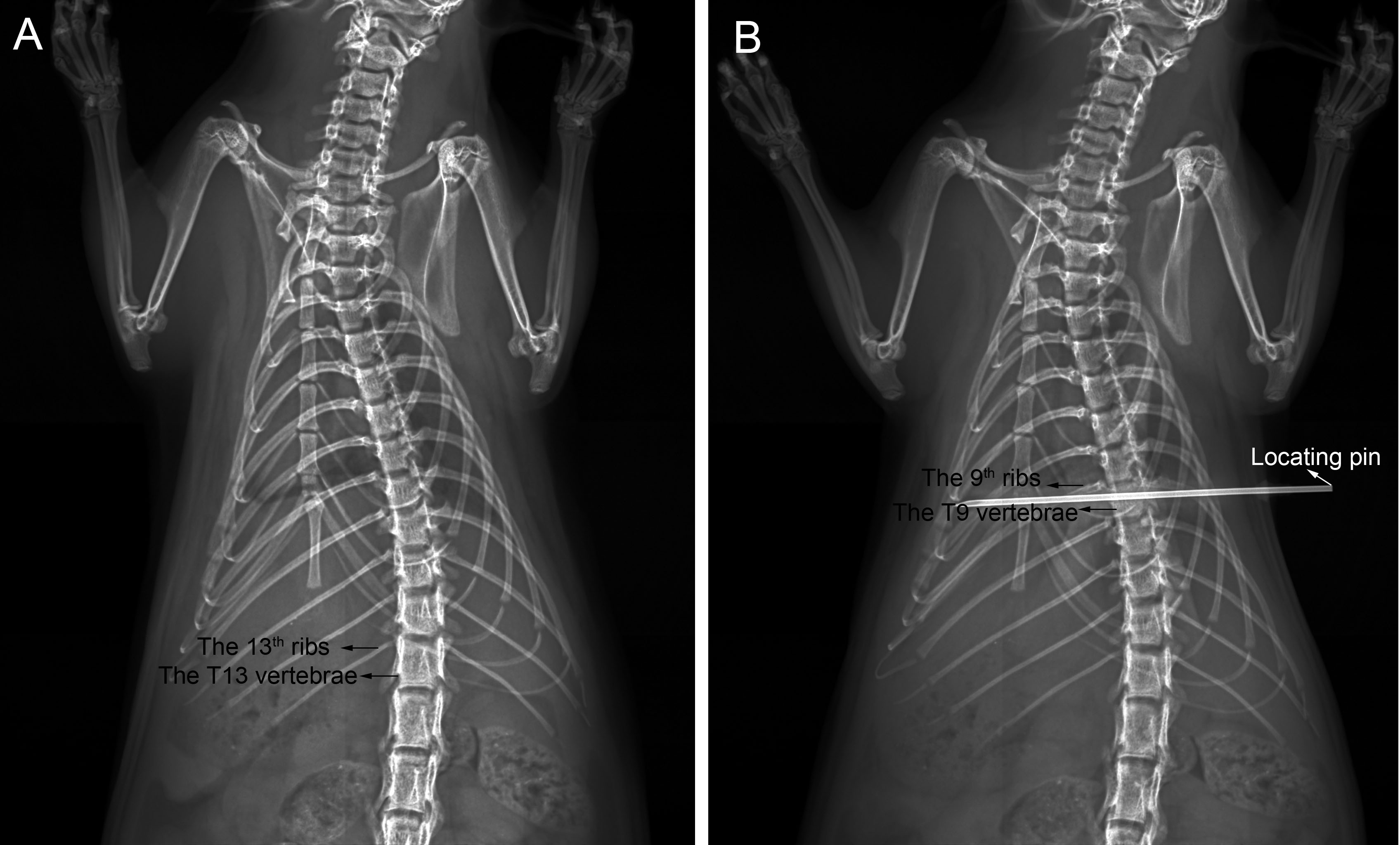
**Figure.S1 The T9 vertebrae of rat was located with a pin in X-ray images.** (A) The 13^th^ ribs and T13 vertebrae were determined in X-ray images, which were taken via the animals digital X-ray machine（Kubtec Model XPERT.8; KUB Technologies Inc）. (B) The T9 vertebrae was located via a locating pin before the procedure of spinal cord injury.
